# Cross Validation of the Gambling Problem Severity Subscale of the Canadian Adolescent Gambling Index (CAGI/GPSS) on a Sample of Ontario High School Students

**DOI:** 10.1007/s10899-017-9731-1

**Published:** 2017-11-28

**Authors:** Nigel E. Turner, Tara Elton-Marshall, Jing Shi, Jamie Wiebe, Angela Boak, Mark van der Maas, Robert E. Mann

**Affiliations:** 1 Institute for Mental Health Policy Research, Centre for Addiction and Mental Health, 33 Russell Street, Toronto, ON M5S 2S1 Canada; 2 Dalla Lana School of Public Health, University of Toronto, Toronto, ON Canada; 3 Department of Epidemiology and Biostatistics, Western University, London, ON Canada; 4 Rehabilitation Science Institute, University of Toronto, Toronto, ON Canada; 5 Responsible Gambling Council, Toronto, ON Canada

**Keywords:** Adolescent gambling, Problem gambling, Psychometrics properties, School survey

## Abstract

This paper reports on the cross validation of the *Gambling Problem Severity Subscale* of the *Canadian Adolescent Gambling Index* (CAGI/GPSS). The CAGI/GPSS was included in a large school based drug use and health survey conducted in 2015. Data from students in grades 9–12 (ages 13–20 years) derived from the (N = 3369 students). The CAGI/GPSS produced an alpha of 0.789. A principle component analysis revealed two eigenvalues greater than one. An oblique rotation revealed these components to represent consequences and over involvement. The CAGI/GPSS indicated that 1% of the students fell into the “red” category indicating a severe problem and an additional 3.3% scored in the “yellow” category indicating low to moderate problems. The CAGI/GPSS was shown to be significantly correlated with gambling frequency (r = 0.36), largest expenditure (r = 0.37), sex (more likely to be male) (r = −0.19), lower school marks (r = −0.07), hazardous drinking, (r = 0.16), problem video game play (r = 0.16), as well as substance abuse. The CAGI/GPSS was cross validated using a shorted version of the short SOGS, r = 0.48. In addition the CAGI/GPSS and short SOGS produced very similar patterns of correlations results. The results support the validity and reliability of the CAGI/GPSS as a measure of gambling problems among adolescents.

## Introduction

Adolescents are often viewed as being particularly vulnerable to gambling-related harms (Gupta and Derevensky 1998a; Shaffer et al. 1999; Turner et al. 2008a). Previous studies of gambling among adolescents found that as many as 80% of adolescents engage in some form of gambling and that they participate in a wide variety of gambling activities (Elton-Marshall et al. 2016; Gupta and Derevensky 1998b; Turner et al. 2011, 2008a, b). Given that most commercial gambling has age restrictions, much of the gambling among adolescents takes the form of private bets. Some studies of the prevalence of problem gambling among adolescents have found the rate is two to three times higher than that of adults (Gupta and Derevensky 1998a; Ladouceur 1996; Shaffer and Hall 2001; Shaffer et al. 1999; Turner et al. 2008a; Wiebe et al. 2005). Furthermore, research shows that adults with gambling problems often started to gamble during adolescence (Griffiths 1995; Gupta and Derevensky 1998b; Kaminer and Petry 1999; Shaffer and Hall 1996; Turner et al. 2006).

Reports of higher prevalence of gambling problems among adolescents have been challenged by some researchers (Ladouceur et al. 2000; Welte et al. 2008). In particular, it was argued by Ladouceur et al. (2000) that adolescents endorse items on questionnaires more often because they do not understand the items (Wiebe et al. 2005). Furthermore, Wiebe et al. (2005) noted that the estimated prevalence varies from study to study in part because of differences in the measures and methods used. In addition, these previous measures were either created for adult with gambling problems or were adapted for adolescents (Wiebe et al. 2005). Tremblay et al. (2010) have recently developed a scale that was designed specifically for adolescents, called the *Gambling Problem Severity Subscale* of the *Canadian Adolescent Gambling Index* (CAGI/GPSS). The project was sponsored by the Canadian Centre on Substance Abuse and the Interprovincial Consortium on Gambling Research.

The development of the CAGI/GPSS was undertaken in three phases (Tremblay et al. 2010). The first phase developed a conceptualization and operational definition of problem gambling specific to the adolescent population, as well as a draft pool of items for measuring problem gambling (Wiebe et al. 2005). Phase two pilot tested an English and French version of the pool of items with a sample of adolescents drawn from school populations in Manitoba and Québec. This was followed a year later with a larger sample of 2394 students, a retest of 343 students from the general school survey (Wiebe et al. 2007), and clinical interviews with 109 students who initially participated in the general school survey. Phase three fine-tuned cut scores and further validated the instrument by testing with students at increased risk of having problems with gambling problems (e.g. adolescents who were receiving treatment for substance abuse or were receiving services from youth centres) or who were currently experiencing problems with gambling and were classified as pathological gamblers based on clinician ratings (Tremblay et al. 2010). The CAGI scores were compared to the clinicians ratings (Tremblay et al. 2010).

The final version of the CAGI consists of 24 items covering the consequences of gambling/betting. The 24 items are composed of three subscales related to consequences (psychological, social, and financial), a fourth subscale related to loss of control and a fifth subscale that measures the global severity of gambling problems. The fifth subscale, referred to as the Gambling Problem Severity Subscale (GPSS) consists of items from the three consequences subscales and the loss of control subscale.

As a result of this development process, the CAGI/GPSS was the first problem gambling measure that was developed specifically for adolescents rather than an adoption of existing instruments developed and tested on adults (Stinchfield 2010).^1^ The CAGI/GPSS is similar to the Canadian Problem Gambling Index that was developed by Ferris and Wynne (2001) for adults, in that it is designed to provide a continuum of problem gambling severity (Stinchfield 2010) from non-problem gambling to high risk problem gambling. The nine-item Gambling Problem Severity Subscale (GPSS) of the CAGI is designed to categorize gambling into “no problem gambling,” “low to moderate severity,” and “high severity.” Although the scale is based on a solid research foundation (Edgren et al. 2016), there is currently a lack of research cross validating the psychometric properties of this scale, comparing the scale to an existing measure of gambling problems, and few studies have examined the factors associated with problem gambling as measured by this scale.

The current study examines problem gambling among a representative sample of adolescents in the province of Ontario, using data from the 2015 Survey Ontario Student Drug Use Heath Survey (OSDUHS; Boak et al. 2015). This is one of the first studies to use the CAGI/GPSS in a general population survey of adolescents. This study therefore provides a valuable opportunity to test the internal validity and external validity of the CAGI/GPSS and to compare the CAGI/GPSS to another measure of problem gambling among youth (the Short South Oaks Gambling Screen).

## Methods

### Sample

Data from students in grades 9–12 (ages 13–20 years) derived from the 2015 cycle of the OSDUHS survey were analyzed. The OSDUHS, conducted every 2 years since 1977, is funded by the Ontario Ministry of Health and Long Term Care and is the longest ongoing school study of adolescents in Canada. This cross-sectional, anonymous in-class survey, which employs a regionally-stratified, two-stage cluster (school, class) sampling design, monitors substance use, mental and physical health, and risk behavior among students in grades 7–12 in Ontario. The 2015 cycle was based on a total sample of 10,426 pupils in 220 publicly funded elementary/middle and secondary schools. The gambling problem scales were contained in half the questionnaires developed for high school students only (grades 9–12), which were randomly distributed within each classroom resulting in a sample of 3426 high school students. Of these, 57 (1.7%) were excluded because of missing information on measures used in this study, resulting in a final sample size of 3369 students.

The questionnaires were administered by staff from the Institute for Social Research, York University on a classroom basis. Students recorded their responses directly onto the questionnaire forms and were instructed not to write their names on the forms. The student participation rate was 60% for high school students. Reasons for student non-completion included absenteeism (11%) and absence of parental consent (29%). The questionnaires were administered between November 2014 and June 2015. Institutional research ethics committees at Centre for Addiction and Mental Health, York University, as well as at 30 district school boards approved this study. Further study details are provided in  Boak et al. (2015).

### Measures

#### Problem Gambling

Problem gambling was measured using two scales. The Gambling Problem Severity Subscale from the *Canadian Adolescent Gambling Index* (CAGI/GPSS) and a shortened version of the South Oaks Gambling Screen Revised for Adolescents (SOGS-RA), referred to as the short SOGS. The GPSS consists of nine items (see Table 1) scores on 4 point scale from 0 to 3, and then a total score was computed (see note in Table 1). The nine CAGI/GPSS items were scored from 0 to 3, for a total score ranging from 0 to 27 (Tremblay et al. 2010). The total score was then categorized as 0–1 = No problem (green light), 2–5 = a Low‐to‐moderate severity (yellow light), and 6+ = High severity (red light) (Tremblay et al. 2010). Never gambled and never gambled in past 3 months were coded as 0. The short SOGS was created from the SOGS-RA due to space limitations in the survey several years ago. The short SOGS is a list of six gambling symptoms that were taken from the SOGS-RA (Winters et al. 1993). Six items were selected to maximize the content and variance of the full SOGS-RA with a minimum of items (Adlaf and Paglia-Boak 2003; Cook et al. 2015). The items were each scored as 1 for yes and 0 for no for a total score ranging from 0 to 6. The short SOGS has a coefficient alpha measuring internal consistency of alpha = 0.71 (Adlaf and Paglia-Boak 2003). Gambling activities were measured by a series of 11 questions about frequency of participation in various gambling activities and one question on overall expenditure.

**Table 1:** Gambling Problem Severity Subscale from the Canadian Adolescent Gambling Index (CAGI/GPSS) severity scale items-total statistics for CAGI/GPSS items

In the last 3 months…	Item-total correlation	Alpha if item deleted
(1) How often have you skipped practice or dropped out of activities (such as team sports or band) due to your gambling	0.422	0.776
(2) How often have you skipped hanging out with friends who do not gamble to hang out with friends who do gamble?	0.574	0.762
(3) How often have you planned your gambling activities?	0.327	0.813
(4) How often have you felt bad about the way you gamble?	0.600	0.755
(5) How often have you gone back another day to try to win back the money you lost while gambling?	0.616	0.747
(6) How often have you hidden your gambling from your parents, other family members, or teachers?	0.520	0.766
(7) How often have you felt that you might have a problem with gambling?	0.643	0.755
(8) How often have you taken money that you were supposed to spend on lunch, clothing, movies, etc., and used it for gambling or for paying off gambling debts?	0.526	0.765
(9) How often have you stolen money or other things of value in order to gamble or to pay off your gambling debts?	0.409	0.783

For items 1–7 the scale was scored as follows: 0 = Never, 1 = Sometimes, 3 = Most of the time and 3 = Almost always. For items 8 and 9, the scale was as follows, 0 = Never, 1 = 1–3 times, 2 = 4–6 times, and 3 = 7 or more times. The total score was the sum of all items responses. The wording of the items has been altered somewhat by removing the words bet and betting from the items to make it easier to read

#### Substance Use and Abuse

Past year cigarette smoking was recorded if the student smoked at least one cigarette daily or smoked occasionally during the past 12 months (Paglia-Boak et al. 2011). Students who smoked a few puffs or less than one cigarette in the past 12 months were not classified as smokers (binary coded as 1 = smokers, 0 = non-smokers). Past year alcohol use was recorded if the student reported that they consumed any alcohol during the past 12 months (Paglia-Boak et al. 2011). Students were asked if they used cannabis at least once during the past 12 months (binary coded as 1) (Paglia-Boak et al. 2011).

To measure substance problem use, the questionnaire included the 6-item CRAFFT^2^ screener that assesses drug use problems experienced by adolescents (Knight et al. 2002). The six yes/no items pertain to problems experienced during the past year. Those endorsing two or more symptoms (binary coded as 1) identified adolescents as having a drug use problem. Hazardous and harmful drinking was used using the Alcohol Use Disorders Identification Test (AUDIT), which was developed by the World Health Organization (Saunders et al. 1993). This instrument is designed to detect problem drinkers at the less severe end of the spectrum of alcohol problems, and has been used in several previous studies (e.g. Adlaf and Ialomiteanu 2000; Turner et al. 2011). Those with a score of eight or more (out of 40) are considered to be drinking at a hazardous or harmful level. The reliability coefficient (a) for these items is 0.87.

#### Sociodemographic and Other Correlates

Sociodemographic correlates included: sex, grade, ethno-racial background, self-estimated current average school marks (Paglia-Boak et al. 2011) and problem video game play assessed using The Problem Videogame Playing (PVP) scale (Tejeiro Salguero and Morán 2002).

### Data Analysis

Descriptive analyses of the CAGI/GPSS were used to calculate the reliability, component structure, and correlates of the GPSS. In addition we computed an estimate of the prevalence of moderate and severe problems (yellow and red) assuming the cut off points recommended by Tremblay, et al. (2010). In addition, Chi-square analyses were used to examine GPSS scores by sex, grade, ethno-racial background, and school grades. All analyses were conducted using Stata 11.0 (StataCorp 2009).^3^

## Results

In total 3369 participants completed the CAGI questionnaire; 26.8% in Grade 9, 25.8% in Grade 10, 23.6% in Grade 11, and 23.8% in Grade 12. The sample was 54.4% female and 45.6% male. The majority of students (58.6%) estimated their grade average to be in the A range (80–100%). For ethnic self-identification, White was the most popular category at 69.9% followed by one of the East or Southeast Asian categories (12.8%), South Asian (7.5%), Black (7.5%), Aboriginal/First Nations (4.0%), Latin American (3.8%), West Asian/Arab (3.4%) and “not sure”(1.4%).

### Gambling Problem Severity Subscale of the CAGI

The Gambling Problem Severity Subscale (CAGI/GPSS) consists of nine items shown in Table 1. The reliability of these items is good with an alpha of 0.789. As shown in Table 1 the item total correlations ranged from 0.33 to 0.53; the alpha if deleted suggested improving the alpha score if one item was deleted, but only slightly to 0.81 from 0.79.

**Fig. 1:**
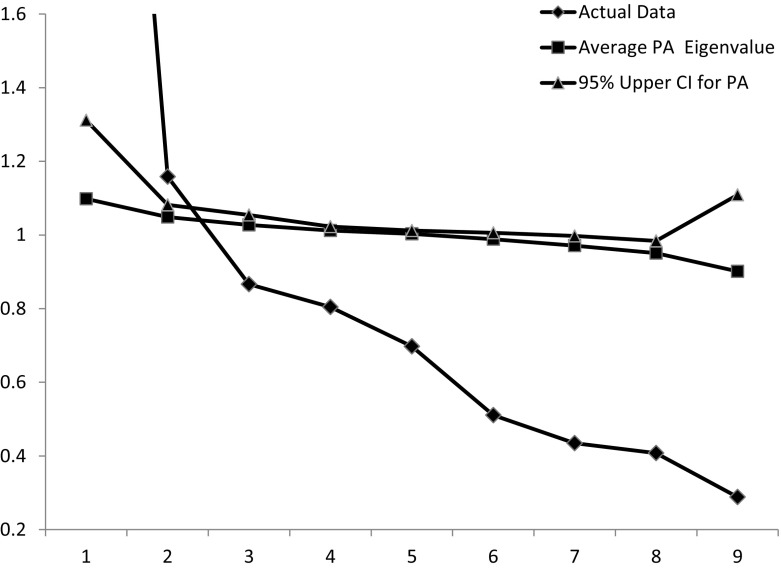
Distribution of Eigenvalues for actual data and the average eigenvalue from the parallel analysis (PA) as well as the 95 percentile from the parallel analysis

In addition, we examined the CAGI/GPSS items using component analysis. The first eigenvalue was 3.83 which accounts for 42.5% of the variance. The second eigenvalue was 1.16 which is larger than 1.0, suggesting that the eigenvalue represents a small but significant second component. To test if the scale was in fact unifactorial we computed 60 parallel analysis simulations based on the actual data set (see Horn 1965; Turner 1998). These simulated data sets were created by scrambling the actual variables in order to test the chances that the second eigenvalue would be as large if the data was in fact random. Figure 1 shows the observed eigenvalues, as well as the average parallel analysis eigenvalue and the 95th percentile for the parallel analysis eigenvalues. As noted above the second eigenvalue was 1.16. The second eigenvalue of the parallel analysis was 1.0466 with a standard deviation of SD = 0.0178. The 95% upper confidence level for the second eigenvalue was computed to be 1.0815 which is well below the size of the second observed eigenvalue in the real data, 1.16, indicating that the second eigenvalue represents real common variance.

Both varimax and oblimin rotations were conducted for the two components. These two analyses produced very similar components, but the oblimin was somewhat cleaner. The oblimin (delta = 0.1) rotation is shown in Table 2. The correlation between the components was rho = 0.55. The first component had the highest loadings on item 2 (skipped hanging out with friends), item 9 (stolen money) and item 1 (skipped practice or dropped out of activities) and could be interpreted as representing consequences of problem gambling. The second component has the highest loadings on item 6 (hiding gambling from family), item 5 (trying to win back money) and item 1 (skipped practice or dropped out of activities) and could be interpreted as a component representing over involvement in gambling. Item 7 (felt you might have a problem with gambling) had nearly equal weak loadings on component 1 (0.31) and component 2 (0.28). Item 8 (spending lunch money gambling) only had a weak loading of 0.30 on component 1. Note that removing the item with the lowest item total correlation, item 3 (see Table 1), did not alter the factor structure.

**Table 2:** Oblimin rotation of CAGI/GPSS items

	Factor 1	Factor 2
Component 1: consequences		
(2) How often have you skipped hanging out with friends who do not gamble/bet to hang out with friends who do gamble?	0.53	0.01
(9) How often have you stolen money or other things of value in order to gamble/bet or to pay off your gambling debts?	0.51	− 0.08
(1) How often have you skipped practice or dropped out of activities (such as team sports or band) due to your gambling?	0.48	− 0.04
(7) How often have you felt that you might have a problem with gambling?	0.31	0.28
(8) How often have you taken money that you were supposed to spend on lunch, clothing, movies, etc., and used it for gambling/betting or for paying off gambling debts?	0.30	0.19
Component 2: over involvement		
(6) How often have you hidden your gambling from your parents, other family members, or teachers?	− 0.06	0.53
(5) How often have you gone back another day to try to win back the money you lost while gambling?	0.01	0.52
(3) How often have you planned your gambling activities?	− 0.12	0.43
(4) How often have you felt bad about the way you gamble?	0.16	0.38

These loadings are computed by Stata 11.0 (StataCorp 2009) which presents them differently than SPSS. In Stata the sum of all squared loadings equal 1.0 whereas in SPSS the sum of all squared loading equal the eigenvalue. As a result these weights in Stata are smaller than the equivalent weights in SPSS (e.g. a Stata loading of 0.5 could be equivalent to an SPSS loading of 0.7 or 0.8)

Table 3 presents the means, standard deviations, and endorsement patterns for the 9 CAGI/GPSS items (coded from 0 = never to 3 = always). An examination of the individual items indicates that the items vary from a mean of 0.060 for item 3 to a low of 0.007 for item 9. In particular 24 students endorse “always” for time 3, whereas only one student endorsed “always” for item 9. Additionally, 3 of the 4 most frequently endorsed items (items 3, 5, and 6) are loaded on factor 2, “over involvement” and the 4 least frequently endorsed items are all from factor 1, “consequences”. This suggests items related to over involvement for the most part have a lower threshold for response then those items related to consequences.

**Table 3:** CAGI/GPSS items means, standard deviations, and response distribution

Item	*N*	M	*SD*	Never (0)	Sometimes (1)	Often (2)	Always (3)
1	3367	0.018	0.205	3336	12	8	11
2	3368	0.014	0.166	3337	20	5	6
3	3368	0.060	0.334	3235	88	21	24
4	3365	0.020	0.182	3316	36	8	5
5	3357	0.036	0.245	3268	67	12	10
6	3357	0.035	0.282	3293	30	13	21
7	3358	0.014	0.158	3326	23	4	5
8	3356	0.020	0.178	3304	40	8	4
9	3356	0.007	0.108	3340	10	5	1

As expected, the distribution of scores on the CAGI/GPSS was highly skewed (skewness = 9.02) with most of the adolescents not endorsing any of the items, and only a small number endorsing enough to qualify as having a gambling problem. The observed scores ranged from 0 to 19. The distribution of scores is shown in Table 4.

**Table 4:** The distribution of the CAGI/GPSS scores

Score	Frequency	Percent
0.00	3125	92.8
1.00	99	2.9
2.00	41	1.2
3.00	41	1.2
4.00	21	0.6
5.00	9	0.3
6.00	8	0.2
7.00	6	0.2
8.00	2	0.1
9.00	5	0.1
10.00	3	0.1
11.00	1	0.0
13.00	1	0.0
14.00	3	0.1
18.00	1	0.0
19.00	3	0.1
Total	3369	100.0

The severity scoring of the CAGI/GPSS is as follows: 0–1 is considered no problem gambling or “green”, 2–5 is categorized as low to moderate severity or “yellow”, and a score of 6 or more is considered high severity or “red”. Further analysis found that 95.7% of the students scored as no problem with gambling (green), 3.3% reported some problem with gambling (yellow), and 1.0% reported having a severe problem with gambling (red).

### Short SOGS and CAGI/GPSS Comparisons

A reliability analysis was also conducted on the short SOGS that had been used in the survey since 1999. The alpha for the current study was 0.51. The item total correlations were poor compared to the CAGI/GPSS ranging from 0.17 to 0.45. The alpha if deleted measure, did not suggest removing any of the items on the short SOGS.

We next examined the relationship between the CAGI/GPSS and the short SOGS. The correlation between the CAGI/GPSS and the short SOGS was Pearson r = 0.478, Spearman rho = 0.411. The cut off values for the two scales (2 for the short SOGS and 6 for the CAGI/GPSS) produced exactly the same estimate of problem gambling prevalence: 1%. However, there was not a strong overlap in responses to the two scales. As shown in Table 5, only 12 adolescents were identified as problematic with both scales. The absence of a true gold standard makes it impossible to determine which adolescents are actually false positives and which are false negative (e.g. those identified by the short SOGS or those identified by the CAGI/GPSS). Thus the relationship between the short SOGS and the CAGI/GPSS categories is unclear.

**Table 5:** Comparison of CAGI/GPSS categories with the short SOGS

	0 or 1 yes	Yes on 2 or more	Total
1.00 green	3203	11	3214
2.00 yellow	103	9	112
3.00 red	21	12	33
Total	3327	32	3359

To further validate the two measures, we examined the relationship of these two scales to other variables that should be correlated with problem gambling. Spearman correlations were used to examine the relationships because of the highly skewed nature of the variables. As shown in Table 6, both the CAGI/GPSS and the short SOGS were significantly correlated with gambling frequency and largest amount of money spent gambling. The CAGI/GPSS and short SOGS had similar patterns of correlations with the largest bet and with the frequency of participation in various games. In nearly all cases, the correlations for the CAGI/GPSS were somewhat higher than for the short SOGS. The one exception is for betting on dice where the short SOGS correlation is slightly larger than for the CAGI/GPSS. It is interesting that the highest correlations of the CAGI/GPSS are for non-commercial games such as card games and games of skill. However, the exception to this is online forms of gambling.

**Table 6:** Correlations of the CAGI/GPSS and short SOGS, with the largest bet and gambling frequency (*n* = 3267)

Variable	Participation (%)	CAGI/GPSS total	Short SOGS
How often…			
Bet money on cards games	9.8	0.29	0.22
Bet money on dice games	3.0	0.19	0.22
Bet money on games of skill (pool, darts, bowling, chess)	7.9	0.31	0.26
Played bingo for money	5.3	0.13	0.10
Bet money in sports pools	11.0	0.26	0.17
Bought sports lottery tickets	3.0	0.22	0.19
Bought any other lottery tickets	8.6	0.14	0.13
Bet money at video gambling machines	1.8	0.21	0.18
Bet money at casino in Ontario	0.5	0.11	0.08
Bet money over Internet	4.2	0.28	0.21
Bet money in other ways	10.5	0.17	0.17

	Mean (STE)		
Largest amount of money spent gambling	0.45 (0.06)	0.37	0.28
Total gambling frequency	6.18 (0.04)	0.36	0.24

All correlations were significant at *p* < 0.001

As shown in Table 6, the most common gambling activities in the 12 months before the survey were bets on sports pools (11.0%), “other ways” (10.5%), card games (9.8%), and lottery tickets (8.6%). With the exception of lottery tickets, the most common types of gambling games are not commercially controlled types of gambling. The least frequently reported games were bets on casino games in Ontario and video lottery games. This last point is not surprising because casinos have strictly enforced age restrictions. The two commercial games that were played the most often are lotteries and bingo; some of the adolescents are old enough to play these games. Lottery purchases were reported by between 5 and 7% of the students from Grades 9–11, but jumped to 16.8% in Grade 12 by which point many of the students would be legally able to purchase those lottery tickets. Total gambling frequency was computed by adding up the number of times spent playing each of the forms of gambling. As shown in Table 7, total frequency had a correlation of r = 0.36 with the CAGI/GPSS total score and r = 0.24 with the short SOGS. The students in the green category on the CAGI/GPSS reported gambling on average 2.6 times (SD = 15.8). The students who fell into the yellow reported gambling 25.5 times (SD = 55.4), and those in the red category reported gambling 63 times (SD = 160.1) on average. Largest amount spent gambling was correlated with the CAGI/GPSS scores, r = 0.37 and r = 0.28 with the short SOGS. Although 6 out of the 29 youth in the red category reported betting more than $200, 7 reported that their largest bet was less than $10. In addition, more than half of those who reported spending $200 in a single event, scored in the Green zone on the CAGI/GPSS. Table 7 shows a breakdown of largest bet by problem gambling category.

**Table 7:** Largest amount bet by CAGI/GPSS category

	Green	Yellow	Red	
Never gambled in lifetime	1899	8	2	1909
Did not gamble/12 months	239	3	1	243
$1 or less	167	6	2	175
$2–$9	450	28	5	483
$10–$49	287	40	7	334
$50–$99	41	11	6	58
$100–$199	12	7	3	22
$200 or more	15	6	6	27
	3111	109	32	3251

Table 8 displays the correlation of the CAGI/GPSS and the short SOGS as well as giving a detailed breakdown of the CAGI/GPSS problem gambling categories and demographic variables. Males were more likely than females to score in the red and yellow category of the CAGI/GPSS. Younger students were slightly more likely to score in the “green” category compared to older students. Similarly, there was a weak relationship between ethno-cultural background and problem gambling. Proportionately more of the non-white students fell into the red category than white students 1.6 versus 0.7%, the reverse was true for the yellow category 2.4 versus 3.7%. Finally, problem gambling was higher amongst students who reported lower marks in school where only 0.3% of students who report getting mostly As, but 9.8% who reported getting mostly Ds, were categorized as “red”. Table 8 also provides the Spearman correlations for these variables with the CAGI/GPSS and short SOGS. Sex and school marks are linearly correlated with both the CAGI/GPSS and short SOGS, but the linear component for white versus other and for grade were not significant.

**Table 8:** Demographic variables and CAGI/GPSS categories

Variables	CAGI/GPSS categories			Total	Correlations	
	Green (%)	Yellow (%)	Red (%)		CAGI/GPSS	Short-SOGS
Sex						
Male	92.1	5.9	1.9	1536	− 0.19***	− 0.12***
Female	98.7	1.1	0.2	1833		
Grade level						
Grade 9	96.4	2.9	0.8	903	0.04*	0.01
Grade 10	96.4	2.2	1.3	869		
Grade 11	94.5	4.8	0.6	795		
Grade 12	94.9	3.6	1.2	802		
White						
No	96.0	2.4	1.6	1014	0.03	0.00
Yes	95.6	3.7	0.7	2355		
School marks						
Mostly A+: 90–100%	97.2	2.7	0.2	528	0.07***	0.10***
Mostly As: 80–89%	96.5	3.1	0.3	1443		
Mostly Bs: 70–79%	94.9	3.7	1.4	1127		
Mostly Cs: 60–69%	92.9	4.5	2.7	225		
Mostly Ds and Fs: 59% and below	87.8	2.4	9.8	37		

Sample size varies slightly from question to question due to missing values. Only 3 students reported grades of F, so they have been combined with those who reported getting mostly D’s; *p < 0.05; ***p < 0.001

In addition, we explored variables that have been shown to be related to problem gambling in previous research. It is well known that people who report one type of addiction are also more likely to report having problems with other addictive behaviors. As shown in Table 9, both the total CAGI/GPSS score and the short SOGS are weakly correlated with a number of other addictive behaviors. Spearman correlations were used due to the skewed nature of these variables. The highest correlations are for the GPSS with the AUDIT, rho = 0.16, and the GPSS with the PVP rho = 0.16. The pattern of correlations is very similar for the two measures of gambling problems, but the correlations for the CAGI/GPSS total are slightly higher likely due to its superior reliability. For example, the AUDIT has correlations of rho = 0.16 with the GPSS and rho = 0.13 with the short SOGS. The similarities in the correlations indicate that the two measures are parallel. These correlations are weak correlations, but are consistent with the general findings in the literature.

**Table 9:** Spearman correlations of the CAGI/GPSS total score with other measures of addiction problems

Variable	CAGI/GPSS total	Short SOGS
CRAFFT scale sum score measuring substance abuse	0.14	0.13
AUDIT scale sum score measuring hazardous drinking	0.16	0.13
Smoked tobacco cigarettes in the past 12 months	0.13	0.09
Drank alcohol in the past 12 months	0.11	0.08
Used cannabis (marijuana or hashish) at least once in the past 12 months	0.11	0.10
Problem video game play (PVP)	0.16	0.14

All correlations are significant at the *p* < 0.001 level

## Discussion

The CAGI/GPSS is the first problem gambling scale designed specifically for adolescents (Tremblay et al. 2010) and therefore has the potential to be adopted for research examining adolescent problem gambling. However, prior to wide adoption there is a need for evidence demonstrating the validity and reliability of the scale and demonstrating whether the scale is superior to existing measures used for evaluating adolescent problem gambling. This study demonstrated that the CAGI/GPSS has an adequate level of reliability and in general the psychometric properties of the CAGI/GPSS are promising. The Cronbach alpha was 0.79 which is high. The item analysis indicated that the items had strong item total correlations. However, the alpha if deleted did indicate that if item 3 was deleted, the scale would have a slightly higher reliability of 0.813. This is in spite of the fact that the item had a respectable item total correlation of 0.33. The item in question was “How often have you planned your gambling activities?” This item attempts to measure preoccupation may be somewhat ambiguous because planning gambling does not necessarily mean excessive gambling. For example, someone making sports bets, will study the teams past performances in order to determine which team to bet on. Such behavior is not necessarily problematic. Thus item 3 may have skewed the results of the severity scale towards people who participate in games of skill. But at the same time young male problem gamblers most often are in fact playing games of skill or betting on sports, and many overestimate their level of skill in these games.

Overall, the pattern of findings suggests that adolescents in the “low to moderate” and “high severity” categories are distinct therefore lending further credibility to the utility of the CAGI/GPSS as a measure for detection of problem gambling. In addition, the pattern of findings in terms of demographic variables (e.g. sex, grade) and comorbidities (e.g. alcohol problem use, smoking) is consistent with previous research regarding correlates of problem gambling. In additional the short SOGS and CAGI/GPSS produced similar results with the CAGI/GPSS yielding slightly higher correlations than the ShortSOGS in general. What is surprising is that the CAGI/GPSS and the shortSOGS yielded very similar prevalence estimates in spite of having a relatively small overlap. Only 11 individuals were identified as having a severe problem by both scales. More research is needed in order to determine which scale is more accurately identifying the problem gamblers or if the scale need to be refined to improve its accuracy.

In this study it was found that 1% of adolescents were identified as “high severity” and a further and 3.3% were “low to moderate for a combined total of 4.3% as measured by the CAGI/GPSS. The prevalence figures reported in this study are within the range found for adolescent gambling problems (0.2–12.3%) in a recent review (Calado et al. 2016). Both the short SOGS and the CAGI/GPSS produced an estimate of 1% of the youth who scored in the severe problem range (red light) and of an estimate of 3.3% in the yellow light category. These figures are lower than in previous studies (Gupta and Derevensky 1998a; Shaffer et al. 1999), including a recent study of adolescents (Elton-Marshall et al. 2016) which reported a prevalence of 1.7% severe problem and 3.5% moderate problem gamblers for a combined total of 5.3% (computed by combining figures from Tables 3, 5). However, this study examined problem gambling rates among adolescents in three provinces (Ontario, Newfoundland, and Saskatchewan) whereas the current study examined problem gambling prevalence in Ontario; therefore differences may be attributable to differences in the populations sampled.

Nonetheless, the prevalence figures in the current study are toward the higher end of that previously found for adult problem gambling prevalence which ranges from 2.0 to 3.0% for the combination of moderate and severe problem gambling and from 0.5 to 1.0% for severe problem gambling (Cox et al. 2005; Williams et al. 2012). Thus the results of our study are consistent with previous research demonstrating higher problem gambling prevalence amongst adolescents compared to adults identified in previous studies (Gupta and Derevensky 1998a; Shaffer et al. 1999) but only provides at best weak support for the hypothesis that adolescents are more vulnerable to problem gambling (Gupta and Derevensky 1998b; Turner et al. 2008a). Further study is needed to determine the distribution of CAGI/GPSS scores in the adolescent population.

Additionally, it’s possible that the lower prevalence rate of 1%, which was found consistently for both the CAGI/GPSS and the SOGS-RA may be attributable to an actual decline in the prevalence of problem gambling. Cook et al. (2015) using 2009 data, reported the short SOGS indicated a prevalence of problem gambling of 2.8%. The decrease in prevalence is consistent with other studies in Canada that have examined the rise and fall of problem gambling prevalence in the country (Williams et al. 2012). Williams et al. (2012) speculate a number of possible reasons for the downward trend including: (a) increased awareness of potential harms; (b) decreased participation because the novelty has worn off; (c) the removal of severe problem gamblers from the population due to severe adverse consequences such as bankruptcy, or suicide; (d) the increased provision of prevention and treatment resources; as well as other factors (Williams et al. 2012, p. 7). Shaffer (2005) has argued that the gradual fall of gambling problem rates is consistent with the theory that a populations tends to adapt over time to the presence of gambling. We would add that for the past few years prevention programs have been run in schools and in addition, an introduction to probability and number sense was added to the school curriculum. The CAGI/GPSS will continue to be used in the OSDUHS to help us monitor trends in adolescent problem gambling over time.

The principle components analysis that was conducted on the data suggests a two factor model of the scale. Ideally a scale should produce only one component which means that the observed variance is explained by a single concept. However the analyses we conducted suggests that the CAGI/GPSS has two components. This is in contrast to the analysis of the CPGI as a measure of problem gambling (Brooker et al. 2009; Loo et al. 2011) which has found it to be unifactorial. However, for the CAGI/GPSS the results suggest that the scale is not unifactorial. If the scale contained more than one component, it would suggest that the concept of problem gambling among youth may not be a single entity. The parallel analyses are particularly important in this respect. The parallel analysis was done with the exact same data, but each variable was independently scrambled for each new analysis. By using a scrambled version of the original data we avoid the problem of distributional assumptions. The parallel analysis (Horn 1965; Turner 1998) clearly shows that an eigenvalue of 1.16 for the second eigenvalue is beyond the 95th percentile for the second eigenvalue with randomly scrambled variables with the same distribution and thus appears to represents a true second component in the data set. In addition, an examination of the loading patterns suggests that the two factors can be interpreted. The oblimin rotation produced two components which we have labelled (1) consequences and (2) over involvement in gambling. An examination of the loadings of the items indicates that in general the over involvement items are more commonly endorsed than the consequence items. This makes sense in terms of the progression of gambling and implies that symptoms of over involvement are the earliest symptoms to emerge, followed by consequences. More research is needed to determine if these components are stable across different samples. In addition, more research should be conducted to explore what “other” games the participants are engaging in.

### Limitations

This study has several limitations common to survey research. Although the sample size is large and the response rate was high for a survey with active consent procedures, the findings are nevertheless subject to possible sample bias. In addition, the findings may reflect some underreporting (faking good) or over reporting (faking bad). The survey did include a false substance as a validity test. Students who endorsed the fictitious drug were removed from the data set. Honest reporting was also encouraged by ensuring confidentiality during data collection. It should also be noted that the cross-sectional nature of the design does not allow for causal inferences regarding the data. Another limitation is that the CAGI has thus far only been used with adolescents. Future research could examine and compare responses between adolescents and youth aged 20–29, with a particular focus on factors of over-involvement and consequences. In particular consequences may be more prominent in slightly older age group, given the length of time that they may have been gambling. Use of both CAGI and CPGI for people in that age group is needed to bridge the gap between youth and adult gamblers.

## Conclusion

The current study supports the validity and reliability of the CAGI/GPSS as a measure of adolescent problem gambling. However, additional research investigating the component structure of the CAGI is needed to determine the replicability of the two component structure found in the current study. In addition, the CAGI/GPSS was correlated with greater involvement in gambling as measured by gambling activities, frequency of play, and largest bet supporting the CAGI/GPSS’s external validity. In terms of validity the CAGI/GPSS produced findings that were consistent with previous measures of problem gambling including higher rates amongst males, higher rates for students with lower school marks, as well as positive correlations with other problems and risk behaviours.

## Footnotes

1 There has since been a scale published in Korean that was developed for adolescents by Park and Jung (2012). Park and Jung’s scale however does not appear to have been calibrated to measure disordered gambling.

2 CRAFFT stands for the 6 items of the scale: Car, Relax, Alone, Forget, Friends, Trouble.

3 The data was collected using a complex design where the schools were randomly selected, and the students were nested within classes that were nested within schools. Typically weights are used due to the unequal probabilities of selection as well as post-stratification weights adjusting for sex and grade to ensure that the sample is representative of the entire provincial student population in publicly funded schools. However, in this paper we are not interested in generalizing to the hypothetical population but are more interested in the quality of the items themselves and the individual participants, so these analyses were not weighted so that each person’s data only represented him or herself.
